# Mechanistic insight into the substrate specificity of 1,2-β-oligoglucan phosphorylase from *Lachnoclostridium phytofermentans*

**DOI:** 10.1038/srep42671

**Published:** 2017-02-15

**Authors:** Masahiro Nakajima, Nobukiyo Tanaka, Nayuta Furukawa, Takanori Nihira, Yuki Kodutsumi, Yuta Takahashi, Naohisa Sugimoto, Akimasa Miyanaga, Shinya Fushinobu, Hayao Taguchi, Hiroyuki Nakai

**Affiliations:** 1 Department of Applied Biological Science, Faculty of Science and Technology, Tokyo University of Science, Chiba, Japan; 2 Department of Applied Life Sciences, Niigata University of Pharmacy and Applied Life Sciences, Niigata, Japan; 3 Graduate School of Science & Technology, Niigata University, Niigata, Japan; 4 Department of Chemistry, Tokyo Institute of Technology, Tokyo, Japan; 5 Department of Biotechnology, The University of Tokyo, Tokyo, Japan

## Abstract

Glycoside phosphorylases catalyze the phosphorolysis of oligosaccharides into sugar phosphates. Recently, we found a novel phosphorylase acting on β-1,2-glucooligosaccharides with degrees of polymerization of 3 or more (1,2-β-oligoglucan phosphorylase, SOGP) in glycoside hydrolase family (GH) 94. Here, we characterized SOGP from *Lachnoclostridium phytofermentans* (LpSOGP) and determined its crystal structure. LpSOGP is a monomeric enzyme that contains a unique β-sandwich domain (Ndom1) at its N-terminus. Unlike the dimeric GH94 enzymes possessing catalytic pockets at their dimer interface, LpSOGP has a catalytic pocket between Ndom1 and the catalytic domain. In the complex structure of LpSOGP with sophorose, sophorose binds at subsites +1 to +2. Notably, the Glc moiety at subsite +1 is flipped compared with the corresponding ligands in other GH94 enzymes. This inversion suggests the great distortion of the glycosidic bond between subsites −1 and +1, which is likely unfavorable for substrate binding. Compensation for this disadvantage at subsite +2 can be accounted for by the small distortion of the glycosidic bond in the sophorose molecule. Therefore, the binding mode at subsites +1 and +2 defines the substrate specificity of LpSOGP, which provides mechanistic insights into the substrate specificity of a phosphorylase acting on β-1,2-glucooligosaccharides.

Glycoside phosphorylases catalyze the phosphorolysis of oligosaccharides into sugar phosphates in the metabolism of specific sugars^1^,^2^. Phosphorylation of sugars without an energy source such as ATP is advantageous for saving energy physiologically^3^. In the application area, the reversibility of reactions and strict regioselectivity of glycoside phosphorylases are of great use for oligosaccharide synthesis^4^. The combination of two glycoside phosphorylases sharing a common donor substrate enhances the use of this type of enzymes, since the use of an expensive sugar 1-phosphate as a starting material can be avoided^5^,^6^. Despite several recent findings regarding new glycoside phosphorylases^7^, the repertoire of glycoside phosphorylases is still much smaller than that of glycoside hydrolases. In addition, the distribution of glycoside phosphorylases is limited to glycoside hydrolase (GH13, GH65, GH94, GH112, and GH130) and glycosyltransferase (GT4 and GT35) families^7^,^8^,^9^ (In the GH3 family, only one glycoside phosphorylase has been reported in subgroup NagZ^10^). Determination of the structural basis for glycoside phosphorylases is important for protein engineering to expand the variety of reactions along with exploration of glycoside phosphorylases.

GH94 along with GH112 exclusively comprises glycoside phosphorylases unlike GH13, GH65, and GH130 and follows inverting mechanism. Cellobiose phosphorylase (CBP)^11^,^12^, cellodextrin phosphorylase^13^, laminaribiose phosphorylase^14^, and 1,2-β-oligoglucan phosphorylase (SOGP, “S” means the initial letter of “sophoro-”oligosaccharide representing “β-1,2-gluco-”oligosaccharide.)^15^, which are members of the GH94 family, share α-d-glucose-1-phosphate (G1P) as a common donor substrate for the synthetic reaction. Recently, cellobionic acid phosphorylase (CBAP), which utilizes gluconic acid as an acceptor substrate, has been reported^16^. The family also contains chitobiose phosphorylase (ChBP) that acts on an chitobiose (GlcNAc-β-1,4-GlcNAc) and produces α-N-acetyl-d-glucosamine-1-phosphate through phosphorolysis^17^. CBP, ChBP and CBAP each form a dimer and show a similar overall quaternary structure, though the amino acid sequence identities between them are relatively low (<30%). They also share the position of the catalytic pocket, which is formed at the dimer interface between the N-terminal domain (Ndom) and the catalytic domain of another subunit, although their acceptor recognition sites are diverse^18^,^19^,^20^. In contrast to these glycoside phosphorylases acting on β-1,4-linked glycosides, no structure of a glycoside phosphorylase acting on β-1,2-glucan and β-1,3-glucan is available.

SOGP is an enzyme given a new EC number (EC 2.4.1.333) according to our recent study on SOGP from *Listeria innocua* (LiSOGP)^15^. LiSOGP phosphorolyzes β-1,2-glucooligosaccharides (alternatively sophorooligosaccharides, Sops) with degrees of polymerization (DP) of 3 or more. The high specificity of LiSOGP to Sop_N_s (N denotes DP) suggests involvement of the enzyme in β-1,2-glucan metabolism together with a β-glucosidase encoded by an adjacent gene^21^,^22^. One of the most remarkable features of LiSOGP homologs is the approximately 250 additional amino acid residues at their N-terminus. This region is supposed to be important for substrate specificity, since it is fully conserved among LiSOGP homologs. However, no LiSOGP homolog has been characterized to date.

*Lachnoclostridium phytofermentans* (formerly, *Clostridium phytofermentans*) is an anaerobic bacterium that is able to metabolize various kinds of plant polysaccharides^23^. The bacterium possesses many genes encoding glycoside phosphorylases including five genes belonging to the GH94 family. One of these genes is an SOGP gene homolog (Cphy_0694, LpSOGP). In this study, we characterized the enzymatic properties of LpSOGP to clarify the features of the SOGP group. Furthermore, we also determined the crystal structure of LpSOGP to understand the structure-function relationship and factors relevant to differences in substrate specificity between other enzymes in the GH94 family.

## Results

### Biochemical characterization of LpSOGP

In the GH94 family, classification based on substrate specificity is almost consistent with phylogenetic grouping^15^. LpSOGP shows 39% amino acid sequence identity with LiSOGP and belongs to the same group as it. We evaluated the general properties of LpSOGP using Sop_2_ as a substrate for the synthetic reaction. The optimum temperature and pH of LpSOGP were 40 °C and pH 7, respectively. LpSOGP was stable in the pH range of 6.5–8.0 (more than 80% residual activity) and up to 37 °C. LpSOGP showed similar *K*_m_ and *k*_cat_ values for Sop_2_, Sop_3_, and Sop_4_, but no significant activity toward Glc in the synthetic reaction (Table 1). Regarding phosphorolytic activity, LpSOGP showed equivalent *K*_m_ and *k*_cat_ values for Sop_3_, Sop_4_, and Sop_5_ to those in the synthetic reaction and showed no activity toward Sop_2_. This kinetic analysis indicates that LpSOGP has essentially the same chain length specificity as to Sop_N_s as LiSOGP. LpSOGP showed sufficiently high *k*_cat_/*K*_m_ values for G1P and inorganic phosphate (Pi) as a GH family enzyme, though the *K*_m_ value for Pi was a little higher than that on LiSOGP. A double reciprocal plot for phosphorolysis of Sop_3_ suggests that LpSOGP follows a sequential Bi Bi mechanism (Fig. S1). LpSOGP showed very weak activity against laminaribiose and no activity toward other disaccharides such as cellobiose or monosaccharides in the synthetic reaction on TLC analysis (Fig. S2A–C). In addition, LpSOGP did not show phosphorolytic activity toward cellobiose or laminaribiose (Fig. S2D). These results indicate that LpSOGP is highly specific to Sop_N_s, as LiSOGP is^15^, and is completely different from other GH94 enzymes in substrate specificity.

### Overall structure

**Figure 1:**
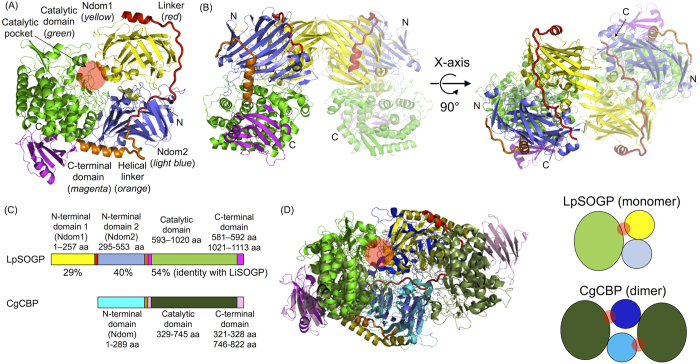
Overall structure of LpSOGP. (**A**) Domain constitution of the LpSOGP monomer. The colors of domains and linkers in LpSOGP are shown in the figure. In the other figures, the same color usage as for LpSOGP is used. Loops in linkers are shown as thick lines. The catalytic pocket is shown as a *red circle* (**A**,**D**). The positions of the N and C-termini are indicated by arrows (**A**,**B**). (**B**) Asymmetric unit of LpSOGP. One subunit in an asymmetric unit is presented semitransparent. (**C**) Comparison of domain constitutions between LpSOGP and CgCBP. The values of percentages indicate the amino acid sequence identity with the corresponding domain in LiSOGP. The N-terminal domain, catalytic domain, C-terminal domain, and helical linker of CgCBP are shown in *cyan, deep green, light pink*, and *light brown*, respectively. (**D**) (*left*) Superimpositioning of the LpSOGP monomer and CgCBP dimer. LpSOGP without Ndom1 and the CgCBP monomer are aligned. The color usage for CgCBP is as in (**C**) except that the N-terminal domains of CgCBP are shown in *cyan* and *blue*. (*right*) Schematic structures of LpSOGP and CgCBP. Linkers and the C-terminal domain are omitted.

The crystal structure of apo LpSOGP was determined at 2.0 Å resolution (Table S1). The crystals contain two identical molecules in an asymmetric unit. The tertiary structure consists of four domains: two N-terminal β-sandwich domains (residues 1–257, *yellow*, Ndom1; and 295–553, *light blue*, Ndom2), a catalytic (α/α)_6_ barrel domain (593–1020, *green*), and a β sheet domain (*magenta*). The β sheet domain consists of a middle segment (581–592) and a C-terminal segment (1021–1113). Ndom2 and the catalytic domain are connected through a helical linker region (554–580, *orange*), and Ndom1 and Ndom2 are connected through a linker composed of a helix and a long loop (258–294, *red*) (Fig. 1A). The helix is included in the linker region due to the alignment with CBAP, ChBP, and CBP. The two molecules in the asymmetric unit are in contact with each other mostly in the Ndom1 region (Fig. 1B). The contact area is only 2.5% (935.5 Å^2^) of the monomer surface area. The estimated Δ*G*^*diss*^ is a negative value (−11.2 kcal/mol), which suggests that the dissociated state is more stable, based on analysis using the protein-protein interaction interface server (PISA, http://www.ebi.ac.uk/msd-srv/prot_int/pistart.html)^24^. In addition, LpSOGP was eluted at the retention time of 100 kDa on size-exclusion chromatography. These results indicate that LpSOGP is a monomeric enzyme, as LiSOGP is^15^.

**Figure 2:**
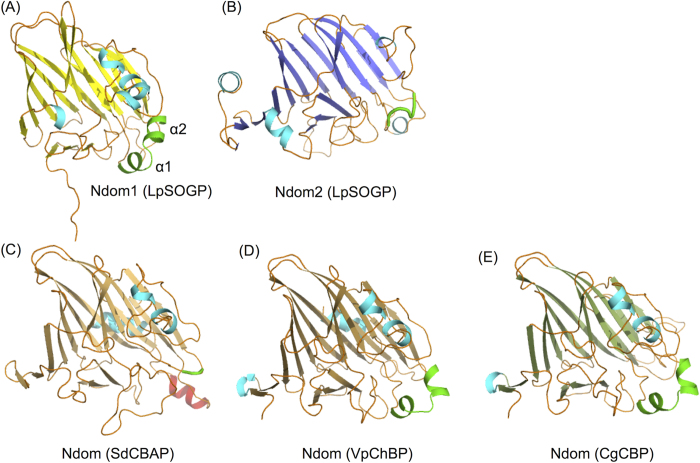
N-Terminal domains of LpSOGP, SdCBAP, VpChBP, and CgCBP. Loops and helices are shown in *orange* and *cyan*. β-Sheets in Ndom1 (**A**), Ndom2 (**B**), Ndoms of SdCBAP (**C**), VpChBP (**D**), and CgCBP (**E**) are shown in *yellow, light blue, light orange, brown, dark green*, respectively. The region important for substrate recognition in Ndom1, and the corresponding positions in Ndom2 and Ndoms are represented in *green*. The helix important for substrate recognition in SdCBAP is shown in *red*.

**Figure 3:**
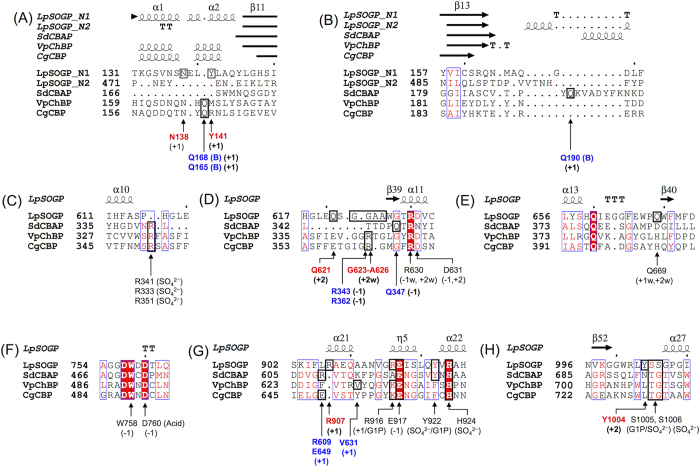
Multiple alignment of GH94 enzymes. Multiple alignment was performed using Matras^43^. Figures were prepared using Espript 3.0 in the ENDscript server (http://espript.ibcp.fr)^44^. (**A**–**B**) Ndom1 and Ndom2 in LpSOGP, and Ndoms in SdCBAP, VpChBP, and CgCBP are aligned. (**C**–**H**) The catalytic domains of LpSOGP, SdCBP, VpChBP, and CgCBP are aligned. Residues related with substrate recognition are labeled, boxed, and indicated by arrows. Residues important for substrate binding at subsites +1 and +2 in LpSOGP are shown in *red bold* letters. *Blue bold* letters represent the residues in SdCBAP, VpChBP, and CgCBP that are located at the positions corresponding to the LpSOGP residues shown in *red bold* letters. Parentheses are the ligands with which the residues interact or the subsites where the residues bind to the ligands. Letters “w” in parentheses indicate that binding to the ligand is mediated by water molecules. (Acid) means that the residue is a catalytic acid. The residues derived from the B subunit are indicated by *bold letter* of B in parentheses.

The overall structure of LpSOGP besides Ndom1 is basically similar to those of GH94 CBAP from *Saccharophagus degradans* (SdCBAP), ChBP from *Vibrio proteolyticus* (VpChBP), and CBP from *Cellvibrio gilvus* (CgCBP) (the RMSD values are 4.11, 3.53, and 3.29 Å, respectively). Ndom1 is unique to LpSOGP, being missing in CBP, ChBP, and CBAP (Fig. 1C). It is interesting that Ndom1 in LpSOGP occupies the same position as the Ndom in subunit B of CgCBP, when subunit A in the CgCBP dimer and the corresponding region of LpSOGP (all except for Ndom1, residues 295–1113) are superimposed (Fig. 1D *left*). This structural observation suggests that LpSOGP forms a catalytic pocket within its tertiary structure unlike the other three GH94 enzymes (Fig. 1D *right*). The structure of Ndom1 is similar to those of both Ndom2 and Ndoms (Fig. 2), though Ndom1 shows low sequence identity with them (<15%) (Table 2). It should be noted that Ndoms in CgCBP, VpChBP, and SdCBAP are similar to Ndom1 rather than Ndom2 in both the primary and 3D structures (Table 2, Fig. 2). On the contrary, the amino acid sequence identity of Ndom1 in LpSOGP with the corresponding region in LiSOGP (29%) is lower than that of Ndom2 (40%) (Fig. 1C). As for the substrate recognition region, two helices (α1 and α2) in Ndom1 constitute a part of the catalytic pocket as well as in Ndoms in VpChBP and CgCBP (Figs 2A,D,E and 3A). The corresponding region in Ndom2 is a short loop like that of SdCBAP, but the substrate recognition helix of SdCBAP is not conserved in Ndom2 (Figs 2B,C and 3B).

### Complex structures with ligands

**Figure 4:**
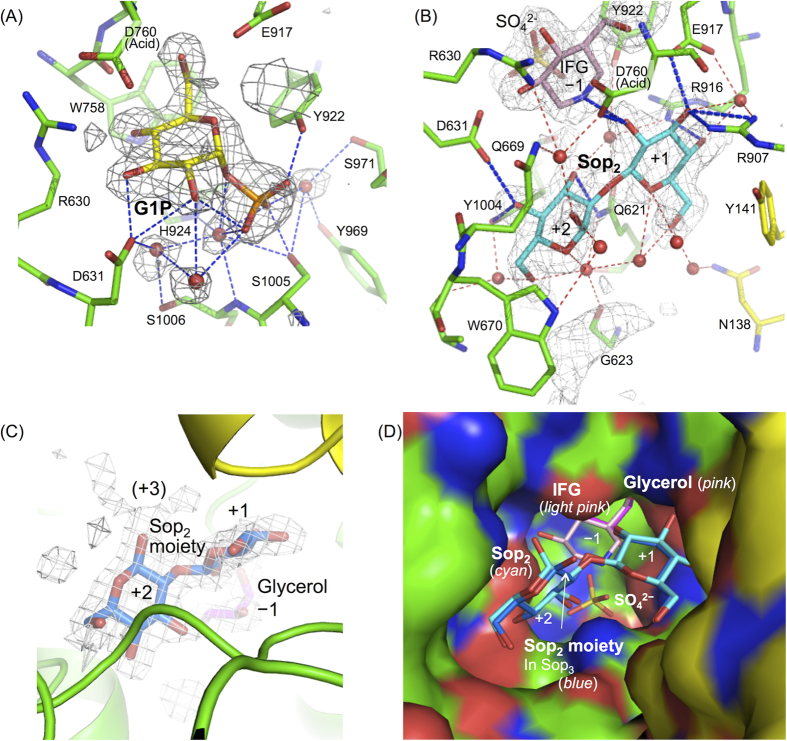
Complex structures of LpSOGP. (**A**–**C**) Electron density maps of G1P (**A**), Sop_2_/IFG/SO_4_^2−^ (**B**), and Sop_3_ (**C**). The *F*_o_−*F*_c_ electron density maps of the ligands are presented as a gray mesh (contoured at 3.0σ). Ligands are omitted for calculation of the *F*_o_−*F*_c_ maps. The ligands and residues important for substrate recognition are shown as sticks. The color usage for LpSOGP follows Fig. 1. Water molecules are shown as *red spheres*. Hydrogen bonds between ligands and residues are represented by *blue dotted lines*. Hydrogen bonds that water molecules participate in Sop_2_ recognition are shown as *red dotted lines* in (**B**). The positions of subsites are shown. The parentheses in (**C**) indicate that the position of subsite +3 is putative. G1P (**A**), Sop_2_ (**B**), IFG (**B**), the Sop_2_ moiety (**C**), and glycerol are shown in *yellow, cyan, light pink, blue*, and *pink*, respectively. (**D**) Ligands in a catalytic pocket. The structures in (**B**) and (**C**) are superimposed and the structure in (**B**) is shown as a surface representation.

**Figure 5:**
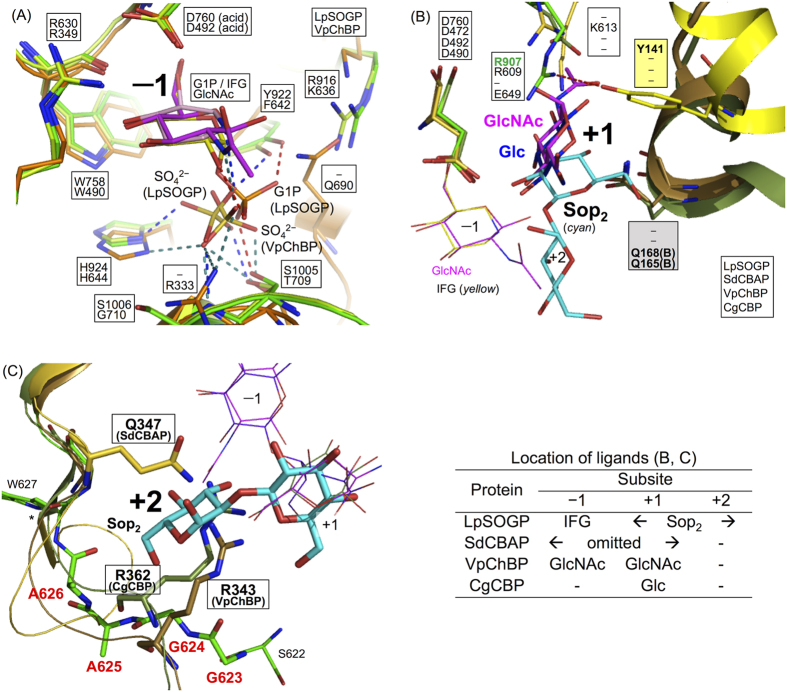
Comparison of subsites −1 (**A**), +1 (**B**) and +2 (**C**). Proteins are represented as cartoons. Glc in CgCBP, and GlcNAc in VpChBP, SdCBAP, VpChBP and CgCBP are shown in *blue, magenta, light brown, brown*, and *dark green*, respectively. The color usage for the other components follows previous figures. Labeled residues, ligands in (**A**), GlcNAc at subsite +1 and Glc in (**B**), and Sop_2_ are shown as sticks. The other ligands are shown as lines. Hyphens in square boxes indicate that there is no corresponding residue. (**A**) Ligands except at subsite −1 are omitted. Hydrogen bonds formed by the phosphate moiety in G1P, SO_4_^2−^ molecules in LpSOGP and VpChBP are represented in *red, blue*, and *green dotted line*. (**B**) Hydrogen bonds between R907 and Y141 are shown as a *red dotted line*. Ligands in SdCBAP are omitted. (**C**) Residues without a boxed label are derived from LpSOGP. The region important for formation of subsite +2 in LpSOGP is shown as a stick and residues in the region are labeled in *red bold* letters. An asterisks indicates that the region beyond the asterisk is omitted for visibility. The table shows the locations of ligands in (**B**) and (**C**).

In order to understand the substrate recognition mechanism, the crystal structures of LpSOGP in complexes with G1P, Sop_2_ (with isofagomine d-tartrate (IFG) and (NH_4_)_2_SO_4_), and Sop_3_ (with (NH_4_)_2_SO_4_) were determined at 2.1, 2.0 and 2.2 Å resolution, respectively (Table S1). In the complex structure with G1P, the electron density of G1P was clearly observed at subsite −1. It is noteworthy that this is the first reported structure of an inverting glycoside phosphorylase complexed with a sugar-phosphate substrate. The Glc moiety in the G1P molecule undergoes direct interactions with R630, D631, W758, D760, and E917 (Figs 3 and 4A), all of which are highly conserved in GH94 enzymes. The position of the Glc moiety is well superimposed with ligands at subsite −1 in other GH94 enzymes such as GlcNAc in VpChBP (Fig. 5A). The phosphate moiety of the G1P molecule forms hydrogen bonds with Y922, S1005, and three water molecules (Figs 3 and 4A). Contrarily, the moiety does not form hydrogen bonds with H924 and S1006 (main chain) that correspond with the reported SO_4_^2−^-recognizing residues (H624 and G710, respectively, in the case of VpChBP). This is because the phosphate moiety is slightly deviated from the position of SO_4_^2−^ in VpChBP and CgCBP (1.1 Å and 1.5 Å, respectively) (Fig. 5A).

Subsites +1 and +2 were revealed by the complex structure with IFG, Sop_2_, and (NH_4_)_2_SO_4_. IFG is located at subsite −1, since IFG is well superimposed with the Glc moiety of G1P (Fig. 5A). In addition, the position of SO_4_^2−^ is almost identical with that in VpChBP. Only the electron density of the α-anomer of Sop_2_ is observed adjacent to IFG (Fig. 4B). The 2-OH group in the non-reducing end glucoside and the 1-OH group in the reducing end glucoside of Sop_2_ face subsite −1 and outside the substrate pocket, respectively. Dihedral angles φ (O5-C1-O1-C’2) and ψ (C1-O1-C’2-C’1) in the Sop_2_ molecule are −69.7° and 146.5°, respectively. The corresponding angles of a free Sop_2_ structure whose energy is minimized^25^ are similar (−72° and 114°, respectively). The conformations of glucosides at both subsites are ^4^*C*_1_. These facts mean that Sop_2_ binds to LpSOGP with only a little distortion, suggesting that Sop_2_ is bound at subsites +1 to +2 productively. Sop_2_ forms 8 hydrogen bonds with Q621, D631, and Y1004 (for binding at subsite +2), and D760, R907, R916, and IFG (for binding at subsite +1) directly. Sop_2_ also undergoes interactions with R630, A626, G623, D760, N138, Q621, and E917 indirectly through the 8 hydrogen bonds with water molecules. Y141 constitutes the substrate pocket by forming a hydrogen bond with R907, though the residue forms no hydrogen bond with Sop_2_.

The Sop_3_ (with (NH_4_)_2_SO_4_)-soaked structure shows clear electron density for the Sop_2_ moiety at subsites +1 and +2 (Fig. 4C). The Glc moiety at subsite +2 is a β-anomer unlike in the case of Sop_2_ soaking, though two ligands at subsites +1 and +2 are well superimposed (Fig. 4C,D), suggesting that Sop_3_ is bound at subsites +1 to +3. However, since weak electron density was observed beyond the 1-OH group of the glucose moiety at subsite +2, Sop_3_ appears to be mostly disordered at subsite +3. The disorder at subsite +3 is perhaps due to fewer hydrogen bond interactions than at subsites +1 and +2. These observations are consistent with the similar kinetic parameters for Sop_2–5_. The electron density of glycerol was observed at subsite −1. The glycerol molecule is well superimposed with a part of IFG at subsite −1 (Fig. 4D). The SO_4_^2−^ ion was not observed in the catalytic pocket perhaps due to precipitation of ligands in the soaking solution. Overall the reaction mechanism of LpSOGP is proposed as shown in Fig. S3.

## Discussion

In this study, we identified the function of LpSOGP, determined its crystal structure, which is the first one among phosphorylases acting on β-linked glucan, and clarified the binding modes of substrates. The similarity between LpSOGP and LiSOGP in enzymatic function and the conserved substrate recognition residues among SOGP homologs (Fig. S4) imply that this group of SOGP homologs shares essentially the same substrate specificity. The structures of LpSOGP enable us to discuss the molecular mechanism of substrate specificity through comparison with those of other structurally available GH94 enzymes. Since many residues related with substrate recognition at subsite −1 and the catalytic acid (D760) are highly conserved among GH94 enzymes spatially (Fig. 5A), these residues were aligned to compare subsites +1 and +2 between LpSOGP and the other enzymes, SdCBAP, VpChBP, and CgCBP (Fig. 3).

The most remarkable difference between LpSOGP, and VpChBP and CgCBP at subsite +1 is the orientation of the ligands (Fig. 5B). Although the Glc moiety in LpSOGP at subsite +1 is located at a similar position to both the GlcNAc molecule in VpChBP and the Glc molecule in CgCBP, the Glc moiety in LpSOGP is rotated by more than 120° about its C2-O2 bond involved in its glycosidic linkage from the molecules in CgCBP and VpChBP. In LpSOGP, this rotation allows the anomeric position of the Glc moiety at subsite +1 to be oriented toward the open space in the catalytic pocket but not toward the wall of the pocket (Figs 4D and 5B). In LpSOGP, R907 protruding to subsite +1 and Y141 forming a hydrogen bond with R907 occupy a position that is able to hinder the binding of the Glc molecule in CgCBP and the GlcNAc molecule in VpChBP sterically. On the other hand, the positions of Q168 (VpChBP) and Q165 (CgCBP) can cause steric hindrance to the Sop_2_ in LpSOGP. The spatial positions of these residues explain the clear difference in substrate binding mode, though these residues correspond in the primary sequences (Fig. 3A). In addition, the positions of these residues depend on the orientations of helices shown in Fig. 5B.

In LpSOGP, the substrate inversion at subsite +1 is likely a key factor for substrate specificity, as described below. The dihedral angle of C1(IFG)-O’2 (Sop_2_)-C’2-C’1 (corresponding to ψ between subsite −1 and +1) is −46.0°, suggesting that the torsion angle of a glycosidic bond between subsites −1 and +1 is obviously different from that of energy-minimized Sop_2_ (114°)^25^ and is unfavorable for substrate binding. In addition, the dihedral angle (ψ) of Sop_2_ in LiBGL, a Sop_2_-degrading β-glucosidase, is also quite different (159.8°)^22^. However, binding of a Glc moiety to subsite +1 itself appears not to compensate for the disadvantage, since no electron density derived from Glc was observed even on soaking with 1 M Glc (data not shown). This is consistent with the fact that the number of direct interactions with Sop_2_ at subsite +1 is fewer than those in other GH94 phosphorylases (Table S2). The disadvantage for substrate binding is likely compensated for at subsite +2, since the glycosidic bond in the Sop_2_ molecule (between subsites +1 and +2) is distorted only a little. Considering the substrate binding of CBP, the dihedral angle of cellobiose (subsites −1 and +1) in CBP from *Cellulomonas uda* (CuCBP) is φ = −82.5° and ψ = 65.0° (PDB ID: 3S4A), being within the range of stable conformation of a free cellobiose molecule^25^. The position and the orientation of the Glc moiety at subsite +1 are similar to those of the Glc molecule in CgCBP. This is consistent with substrate specificity of the CBPs. Overall, the combination of “the inversion at subsite +1” and “the compensation at subsite +2” defines the substrate specificity of LpSOGP.

The architecture at subsite +2 is unique in LpSOGP, compared with in SdCBAP, VpChBP, and CgCBP. The small side chains of Gly and Ala residues provide LpSOGP with sufficient space as subsite +2. On the contrary, Q347 (SdCBAP), R343 (VpChBP), and R362 (CgCBP) protrude to subsite +2 to fill the space and interact with the ligands at subsites −1 and/or +1 (Figs 3D and 5C). In CuCBP, the side chain of the Arg residue corresponding to R362 (CgCBP) is flipped out despite that cellobiose binds to CuCBP^26^. This flipping makes a space corresponding to subsite +2 of LpSOGP, which is consistent with the fact that CuCBP allows gentiobiose (Glc-β-1,6-Glc) and melibiose (Glc-α-1,6-Glc) to be minor acceptor substrates^27^. These observations imply an evolutional relationship between SOGP and other GH94 enzymes.

Ndom1 appears to be generated through domain duplication according to its position and the structural similarity to Ndom2. This duplication monomerizes LpSOGP but retains the fundamental constitution of a catalytic pocket between hetero domains. Such an example is found in isocitrate dehydrogenases^28^. A class II ribonucleotide reductase is another example, though only a part of the domain is duplicated^29^. This evolutional pathway is reversed against oligomerization through swapping of duplicated domains^30^,^31^. Among carbohydrate-active enzymes, the monomeric constitution of GH55 β-1,3-glucanase from *Phanerochaete chrysosporium* composed of tandem β-helical domains seems to be caused by domain duplication^32^,^33^. However, the catalytic domain is located at the interface of the two structurally homologous domains unlike in the case of LpSOGP.

This study clearly provides mechanistic insights into the substrate specificity of LpSOGP, which is an important structural basis for enzymes acting on β-1,2-linked glucosidic bonds. The domain duplication found in LpSOGP is a unique example in carbohydrate-active enzymes and expands the knowledge on molecular evolution.

## Materials and Methods

### Preparation of recombinant LpSOGP

A gene encoding LpSOGP (*cphy_0694*, GenBank^TM^ accession number ABX41081.1) was amplified by PCR from genomic DNA of *L. phytofermentans* as a template using KOD-plus DNA polymerase (Toyobo, Osaka, Japan) with the following oligonucleotides based on the genomic sequence (GenBank^TM^ accession number CP000885): 5′-aaaccatgggcatactaaaaacattgtctg-3′ as the forward primer containing an NcoI site (underlined) and 5′-tttctcgaggttcttaacataaatatg-3′ as the reverse primer containing an XhoI site (underlined). The amplified PCR product was purified using a QIAquick PCR Purification Kit (Qiagen, Hilden, Germany), digested with NcoI and XhoI (New England Biolabs, Beverly, MA, USA), and inserted into the corresponding sites of pET28a (+) (Novagen, Madison, WI, USA) to encode a His_6_-tagged fusion at the C-terminus of the recombinant protein. The expression plasmid was propagated in *Escherichia coli* BL21(DE3) (Novagen), purified using a High Pure Plasmid Isolation Kit (Roche Diagnostics, Mannheim, Germany), and verified by sequencing (Eurofins Genomics K.K., Tokyo, Japan). The transformant was cultured in LB medium containing 30 μg/ml of kanamycin at 37 °C until OD_660_ reached around 0.8. After 0.1 mM IPTG (final concentration) was added to produce recombinant LpSOGP, the cells were cultured at 20 °C overnight. The cells were collected by centrifugation at 3900× *g* for 5 min, suspended in 50 mM MOPS-NaOH (pH 7.5) buffer containing 300 mM NaCl (buffer A), and then disrupted by sonication. The supernatant obtained on centrifugation at 27000× *g* for 10 min was loaded onto a HisTrap FF crude column (5 ml; GE Healthcare, Buckinghamshire, England) equilibrated with buffer A. After the column had been washed with buffer A containing 10 mM imidazole till almost unbound proteins were removed, LpSOGP was eluted with a linear gradient of imidazole (10–300 mM, 55 ml total volume) in the same buffer at a flow rate of 2 ml/min. The eluate was buffered with 20 mM MOPS-NaOH (pH 7.5) using Amicon Ultra 30,000 molecular weight cut-off (Millipore, Billerica, MA, USA) for assaying. For crystallization, the enzyme solution mixed with an equal volume of 50 mM MOPS-NaOH (pH 7.5) containing 60% saturated ammonium sulfate was loaded onto a HiTrap^TM^ Butyl HP column (5 ml; GE Healthcare) equilibrated with 50 mM MOPS-NaOH (pH 7.5) containing 30% saturated ammonium sulfate. The enzyme was eluted with a linear gradient of 30–0% saturated ammonium sulfate (55 ml total volume) in 50 mM MOPS-NaOH (pH 7.5) at a flow rate of 2 ml/min. The eluate was buffered with 5 mM MOPS-NaOH buffer (pH 7.5) using Amicon Ultra 30,000 molecular weight cut-off to concentrate the enzyme to 10 mg/ml. An ÄKTA Prime Plus chromatography system (GE Healthcare) was used for the whole enzyme purification steps. The purity of the enzyme was analyzed by SDS-PAGE using 8% polyacrylamide gels. Protein concentrations were determined from UV absorbance at 280 nm (the extinct coefficient^34^ and theoretical molecular weight of LpSOGP are 167890 cm^−1^M^−1^ and 169640 Da, respectively). For expression of selenomethionine (SeMet)-labeled LpSOGP, the plasmid was introduced into *E. coli* B834(DE3). LeMaster medium containing 30 μg/ml kanamycin was used in place of LB medium. Protein induction and purification were performed in almost the same way as for the native protein.

### Assay

The phosphorolytic activity of LpSOGP was determined by quantifying G1P produced from Sop_N_s and 10 mM inorganic phosphate by coupling assay using phosphoglucomutase and glucose 6-phosphate dehydrogenase^35^. The colorization reagent and substrate solution (85 μl) comprising Sop_N_s, inorganic phosphate, 10 μM glucose-1,6-bisphosphate (Sigma-Aldrich, St. Louis, MO, USA), 10 IU/mL glucose 6-phosphate dehydrogenase from *Leuconostoc sp.* (Oriental Yeast, Tokyo, Japan), 12.5 IU/mL phosphoglucomutase from rabbit muscle (Sigma-Aldrich), 1.0 mM thio-NAD^+^ (Oriental Yeast), 50 mM MgCl_2_, and 100 mM MOPS-NaOH (pH 7.5) was mixed with 85 μl of the enzyme solution in 20 mM MOPS-NaOH (pH 7.5) to start the reaction in a 96-well microplate (EIA/RIA plate, 96-well half area; Corning, NY, USA). The reaction mixture was incubated at 30 °C and the increase in absorbance at 400 nm due to thio-NADH was monitored at 1-minute interval for 10 min with a Spectramax 190 (Molecular Devices, CA, USA).

Synthetic activity was determined by measuring inorganic phosphate produced from acceptors and 10 mM G1P by the Lowry and Lopez method^36^. The substrate solution comprising various concentrations of Sop_N_s and 10 mM G1P in 100 mM MOPS-NaOH (pH 7.0) (140 μl) was mixed with 20 μl of enzyme solution to start the reaction at 30 °C. Aliquots (20 μl) were taken at 2-minute intervals and was mixed with 160 μl of 0.2 M sodium phosphate (pH 4.0) and 25 mM ammonium molybdate containing 25 mM sulfuric acid to stop the reaction. The solutions were mixed with 20 μl of 1% ascorbic acid containing 0.05% potassium sulfate. After the solution had been incubated at 37 °C for 1 h, the increase in absorbance at 700 nm was measured.

For investigation of substrate specificity, the synthetic reaction was performed using a substrate solution containing 10 mM each acceptor substrate and 10 mM G1P in the presence of 100 mM MOPS-NaOH (pH 7.0). Substrates are altered to 10 mM each oligosaccharide and 10 mM inorganic phosphate for phosphorolysis. The concentration of LpSOGP was 0.1 mg/ml when Sop_2_ and Sop_3_ were used as substrates and 1.0 mg/ml for the other substrates. The reaction solution was incubated at 30 °C for 1 h and then the reaction was stopped by heat treatment at 100 °C for 5 min. The reaction products were analyzed by thin layer chromatography (TLC).

### Temperature and pH profiles

The effects of temperature and pH on activity were evaluated as to the synthetic activity using 10 mM Sop_2_ and 10 mM G1P. The optimum temperature and pH were determined by measuring the activity at various temperatures (0–60 °C) and in various pH ranges in 20 mM buffers, respectively, as follows: sodium acetate (pH 4.0–5.5), MES-NaOH (pH 5.5–6.5), MOPS-NaOH (pH 6.5–7.5), Tris-HCl (pH 7.5–9.0), and glycine-NaOH (pH 9.0–10.0). The thermal and pH stabilities were determined from the residual synthetic activity at 30 °C after incubation of LpSOGP (0.5 mg/ml) at various temperatures (0–60 °C) in 100 mM MOPS-NaOH (pH 7.5), and at 37 °C in 20 mM various buffers as described above, respectively.

### TLC analysis

Each reaction solution (0.5 μl) was spotted onto a TLC plate (Kieselgel 60 F_254_; Merck, Darmstadt, Germany). The TLC plates were developed with 75% acetonitrile in water (v/v). The TLC plates were then soaked in a 5% sulfuric acid:95% ethanol (v/v) solution and heated in an oven until bands were sufficiently visible.

### Kinetic analysis

The initial velocities of the synthetic and phosphorolytic reactions with various concentrations of substrates were determined under the standard conditions. The kinetic parameters for Sop_N_s were calculated by curve fitting the experimental data to the Michaelis-Menten equation (1) using GraFit version 7.0.3.


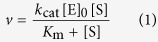


where *v* is reaction velocity, *k*_cat_ is turnover number, *K*_m_ is Michaelis constant, [E]_0_ is enzyme concentration, and [S] is substrate concentration.

### Size-exclusion chromatography

LpSOGP (2 mg/ml) was loaded onto a Superdex^TM^ 200 10/300 GL column (GE Healthcare) equilibrated with 50 mM Tris-HCl (pH 7.5) containing 150 mM NaCl. Ovalbumin (44 kDa), conalbumin (75 kDa), aldolase (158 kDa), ferritin (440 kDa), and thyroglobulin (669 kDa; GE Healthcare) were used as standard proteins. Blue dextran 2000 (2000 kDa; GE Healthcare) was used to determine the void volume of the column.

### Crystallography

All crystals (native and SeMet-labeled protein) used for data collection were obtained at 25 °C using the hanging drop vapor diffusion method by mixing 1–2.4 μl of 10 mg/ml protein solution with an equal volume of reservoir solution comprising 0.1 M Tris-HCl (pH 7.5), 0.2 M calcium acetate, and 10–15% PEG3350. Crystals completely grew in 2–3 days. Crystals were cryoprotected with the reservoir solution containing 30% glycerol for the SeMet-substituted enzyme or 25% PEG400 for the native enzyme. A cryoprotectant containing MgCl_2_ instead of calcium acetate was used to avoid precipitation of ligands when crystals were soaked with soaking solution containing G1P or IFG (Toronto Research Chemicals Inc., Toronto, Canada), Sop_2_, or (NH_4_)_2_SO_4_. IFG was selected as a glucose analog, since it strongly inhibited the synthetic reaction of SOGP in a preliminary experiment. Crystals were then soaked in the cryoprotectants supplemented with 50 mM Sop_3_ (with 50 mM (NH_4_)_2_SO_4_) (SeMet-labeled), 20 mM G1P (native), or 20 mM Sop_2_ (with 1.0 mM IFG and 100 mM (NH_4_)_2_SO_4_) (native). In the case of soaking in 50 mM Sop_3_ (with 50 mM (NH_4_)_2_SO_4_), precipitation in the soaking solution made the concentrations of the ligands obscure. The crystals were cooled and then kept at 100 K in a nitrogen-gas stream during data collection. A set of X-ray diffraction data for each crystal was collected using a CCD detector (ADSC Quantum 210r) on a beamline BL-5A at Photon Factory (Tsukuba, Japan). The diffraction data set was processed using iMosflm^37^ or HKL2000^38^. The initial phase of the LpSOGP apo structure was determined by the single wavelength anomalous dispersion method using AutoSol in Phenix^39^. Automated model building was also performed using the same program. Molecular replacement was performed using MOLREP^40^ to determine the initial phases of complex structures. Manual model building and refinement were performed using Coot^41^ and Refmac5^42^, respectively. Quality checking of the structures was performed using the wwPDB validation server (http://wwpdb-validation.wwpdb.org/validservice/). PyMOL (DeLano Scientific; http://www.pymol.org) was used for the preparation of figures. The buried surface area was calculated with PISA^24^.

## Additional Information

**How to cite this article**: Nakajima, M. *et al*. Mechanistic insight into the substrate specificity of 1,2-β-oligoglucan phosphorylase from *Lachnoclostridium phytofermentans .Sci. Rep.* **7**, 42671; doi: 10.1038/srep42671 (2017).

**Publisher's note:** Springer Nature remains neutral with regard to jurisdictional claims in published maps and institutional affiliations.

## Supplementary Material

Supplementary Information

## Figures and Tables

**Table 1:** Kinetic parameters of LpSOGP for Sop_N_s.

	Synthesis				Phosphorolysis		
	*k*_cat_	*K*_m_	*k*_cat_/*K*_m_		*k*_cat_	*K*_m_	*k*_cat_/*K*_m_
	(s^−1^)	(mM)	(s^−1^ mM^−1^)		(s^−1^)	(mM)	(s^−1^ mM^−1^)
Glc	N.D.^a^				—	—	—
Sop_2_	40 ± 3	5.5 ± 0.9	7.3 ± 0.6		N.D.		
Sop_3_	55 ± 1	2.8 ± 0.3	20 ± 1		47 ± 5	4.8 ± 0.8	9.8 ± 0.6
Sop_4_	46 ± 3	2.2 ± 0.4	21 ± 2		42 ± 3	1.8 ± 0.3	23 ± 2
Sop_5_	—b	—	—		48 ± 3	2.5 ± 0.3	19 ± 1
G1P	28 ± 1	2.6 ± 0.3	11 ± 1	Pi	63 ± 2	11 ± 1	5.7 ± 0.1

Synthetic and phosphorolytic reactions were performed at pH 7.0 and 7.5, respectively.

^a^N.D. indicates that specific activity is less than 0.1% of that for Sop_3_ when 10 mM substrates is used.

^b^− represents not examined.

**Table 2:** Amino acid sequence identity and RMSD among N-terminal domains in GH94 enzymes.

	Enzyme	Domain	Sequence identity (%)				
			LpSOGP		SdCBAP	VpChBP	CgCBP
			Ndom1	Ndom2	Ndom	Ndom	Ndom
				(Cat)	(Cat)	(Cat)	(Cat)
			5H3Z^a^		4ZLF^a^	1V7X^a^	2CQS^a^
RMSD for Cα (Å)	LpSOGP	Ndom1		11	14	14	10
	LpSOGP	Ndom2 (Cat)^b^	3.71		4.6 (18)	7.8 (17)	8.8 (16)
	SdCBAP	Ndom (Cat)	3.03	3.79 (2.77)		20 (26)	13 (25)
	VpChBP	Ndom (Cat)	2.59	3.40 (2.86)	2.53 (2.25)		38 (35)
	CgCBP	Ndom (Cat)	2.60	3.45 (2.62)	2.59 (1.98)	1.35 (1.84)	

The values are based on pairwise alignment using Matras (http://strcomp.protein.osaka-u.ac.jp/matras/)^43^.

The represents 100% for sequence identity and 0 Å for RMSD.

The regions of N-terminal domains and catalytic domains in SdCBAP, VpChBP, and CgCBP are SdCBAP (residues 1–275 and 318–709), VpChBP (1–269 and 310–724), and CgCBP (1–270 and 328–746), respectively.

^a^PDB IDs whose structures were used for preparation of figures and tables.

^b^The values in parentheses are the results calculated on alignment of catalytic domains.
