# Supplementary material for: Mechanistic insight into the substrate specificity of 1,2-β-oligoglucan phosphorylase from Lachnoclostridium phytofermentans
Source: Sci Rep. 2017 Feb 15;7:42671. doi: 10.1038/srep42671 (PMC5309861; doi:10.1038/srep42671)
Supplement: Supplementary Information [file srep42671-s1.pdf]

Title

Mechanistic insight into the substrate specificity of 1,2-β-oligoglucan phosphorylase from *Lachnoclostridium phytofermentans*.

Authors

Masahiro Nakajima[1*], Nobukiyo Tanaka[1], Nayuta Furukawa[1,2], Takanori Nihira[3], Yuki Kodutsumi[1], Yuta Takahashi[3], Naohisa Sugimoto[3], Akimasa Miyanaga[4], Shinya Fushinobu[5], Hayao Taguchi[1], and Hiroyuki Nakai[3]

Affiliations

[1]Department of Applied Biological Science, Faculty of Science and Technology, Tokyo University of Science, Chiba, Japan

[2]Department of Applied Life Sciences, Niigata University of Pharmacy and Applied Life Sciences, Niigata, Japan

[3]Graduate School of Science & Technology, Niigata University, Niigata, Japan

[4]Department of Chemistry, Tokyo Institute of Technology, Tokyo, Japan

[5]Department of Biotechnology, The University of Tokyo, Tokyo, Japan

* Corresponding author

Masahiro Nakajima

E-mail: m-nakajima@rs.tus.ac.jp

Table S1. Data collection and refinement statistics.

Soaked ligands<br>PDB ID	None (Apo)[a]<br>5H3Z	Sop_3, $(NH_4)_2SO_4$[a]<br>5H40	Sop_2, $(NH_4)_2SO_4$, IFG<br>5H41	G1P<br>5H42
**Data collection statistics**				
Space group	*P* 1 2_1 1	*P* 1 2_1 1	*P* 1 2_1 1	*P* 1 2_1 1
Beamline	PF BL-5A	PF BL-5A	PF BL-5A	PF BL-5A
Wavelength (Å)	0.97932	1.0000	1.0000	1.0000
Unit-cell parameters				
a (Å)	87.76	86.78	86.69	86.47
b (Å)	94.78	94.33	94.88	94.78
c (Å)	157.94	156.92	157.36	156.85
β (°)	98.41	97.60	100.81	93.96
Resolution (Å)	50–2.00 (2.03–2.00)	86.02–2.20 (2.24–2.20)	50–2.00 (2.03–2.00)	50–2.10 (2.14–2.10)
Observed reflections	2517987 (122366)	431437 (19944)	623634 (29959)	515663 (25837)
Unique reflections	172867 (8557)	123410 (5677)	168440 (8322)	141346 (6983)
Completeness (%)	100 (100)	96.9 (91.1)	99.7 (99.0)	96.0 (95.3)
R_{merge} (%)	12.1 (86.1)	7.0 (13.6)	12.7 (50.2)	13.4 (55.0)
Mean $I/\sigma(I)$	25.8 (2.8)	9.3 (4.3)	11.0 (1.6)	9.5 (1.8)
Redundancy	14.6 (14.3)	3.5 (3.5)	3.7 (3.6)	3.7 (3.7)
**Refinement statistics**				
Resolution (Å)	42.72–2.00	86.02–2.20	48.27–2.00	48.25–2.10
No. of reflections	164157	117305	160075	134392
R_{factor} / R_{free}	0.168 / 0.204	0.179 / 0.211	0.168 / 0.204	0.195 / 0.236
No. of non-hydrogen atoms				
Protein	17844	17844	17856	17800
Ligand				
Sop_2	-	46	46	-
Glycerol	-	12	-	-
IFG	-	-	20	-
SO_4^{2-}	-	-	10	-
G1P	-	-	-	32
Water	871	741	839	652
Other solvent	84	32	10	28
Average B-factors (Å^2)				
Protein	31.1	27.9	27.6	25.8
Ligand				
Sop_2	-	20.6	26.6	-
Glycerol	-	22.4	-	-
IFG	-	-	22.8	-
SO_4^{2-}	-	-	24.2	-
G1P	-	-	-	24.7
Water	31.1	24.9	26.5	22.8
Other solvent	40.5	37.7	30.1	48.7
r.m.s.d. from ideality				
Bond length (Å)	0.0183	0.0166	0.0169	0.0149
Bond angle (°)	1.67	1.67	1.70	1.68
Chiral volume (Å^3)	0.1131	0.1018	0.1073	0.1006
Ramachandran plot				
Favored region (%)	96.5	96.3	95.8	96.0
Allowed region (%)	3.4	3.6	4.0	3.8
Outer region (%)	0.1	0.1	0.1	0.2

[a] SeMet-labeled enzyme was used.

Values in parentheses are for the outermost shell.

Table S2. Interaction with ligands at subsite +1 in phosphorylases.

	LpSOGP	Kojibiose phosphorylase	VpChBP	CgCBP
PDB ID	5H41	3WIQ	1V7X	2CQS
Ligand at subsite +1	Sop_2	Kojibiose	GlcNAc	Glc
O1	Wat	K596	-	Y653, E649 (2)[a], Q165 (B)[d]
O2	Linkage position	Linkage position	E637 (N)[b] C492[c], V631[c]	E659 (2)[a], Y653, E649
O3	E917, R907 (2)[a]	W391	K636, E637	E659, K658
O4	R916	E392, T417, W391	Linkage position	Linkage position
O5	-	-	Q168 (B)[d]	Q165 (B)[d]
O6	Wat	E392	-	-

[a](2) represents the number of hydrogen bonds.

[b](N) represents interaction with a nitrogen atom in the N-acetyl group.

[c]The hydrophobic interaction with a methyl group.

[d]B in parentheses indicates that the residues are derived from the B subunit.

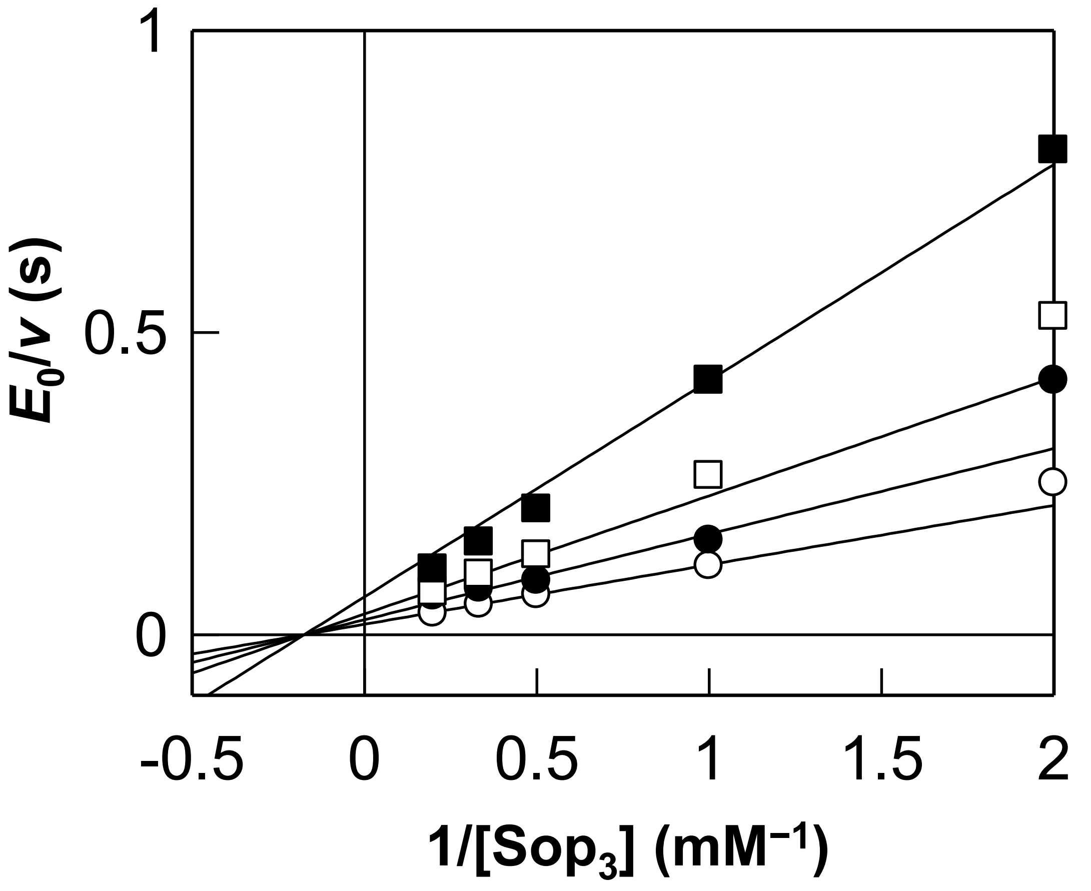

Figure S1. Double reciprocal plot of the phosphorolysis of Sop$_3$ with different concentrations of inorganic phosphate.

The concentrations of inorganic phosphate were 1.0 mM (*closed squares*), 2.0 mM (*open squares*), 3.0 mM (*closed circles*), and 5.0 mM (*open circles*). Grafit version 7.0.3 was used to perform non-linear regression to the following equation for a sequential Bi Bi mechanism, $v = k_{cat}[E]_0[A][B]/(K_{iA}K_{mB}+K_{mA}[B]+K_{mB}[A]+[A][B])$, where v is reaction velocity, k_{cat} is turnover number, K_{mA} and K_{mB} are Michaelis constants for Sop$_3$ and Pi, respectively, K_{iA} is dissociation constant, $[E]_0$ is enzyme concentration, and [A] and [B] are Sop$_3$ and Pi concentrations, respectively. The kinetic parameters are as follows: $k_{cat} = 170 \pm 70$ (s^{-1}), $K_{mA} = 5.7 \pm 4.0$ (mM), $K_{mB} = 9.6 \pm 5.2$ (mM), and $K_{iA} = 5.7 \pm 2.2$ (mM).

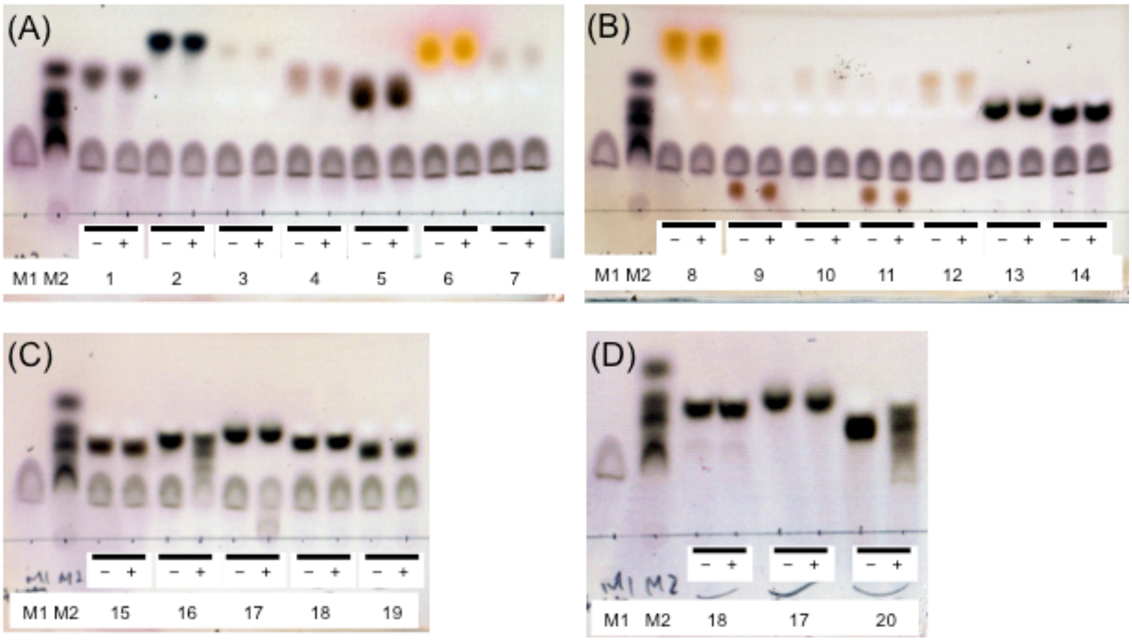

Figure S2. TLC analysis of the products of synthetic and phosphorolytic reactions.

M1, G1P; M2, mixture of glucose and Sop$_{2–5}$. + and − represent with and without an enzyme, respectively. (A–C) Reaction products derived from acceptors and G1P. (D) Reaction products derived from substrates and Pi. The numbers represent glucose (1), 2-deoxy-D-glucose (2), D-xylose (3), D-mannose (4), D-galactose (5), L-fucose (6), L-arabinose (7), L-rhamnose (8), D-glucosamine (9), GlcNAc (10), D-galactosamine (11), N-acetyl-D-galactosamine (12), sucrose (13), maltose (14), lactose (15), Sop$_2$ (16), laminaribiose (17), cellobiose (18), gentiobiose (19), and Sop$_3$ (20), respectively.

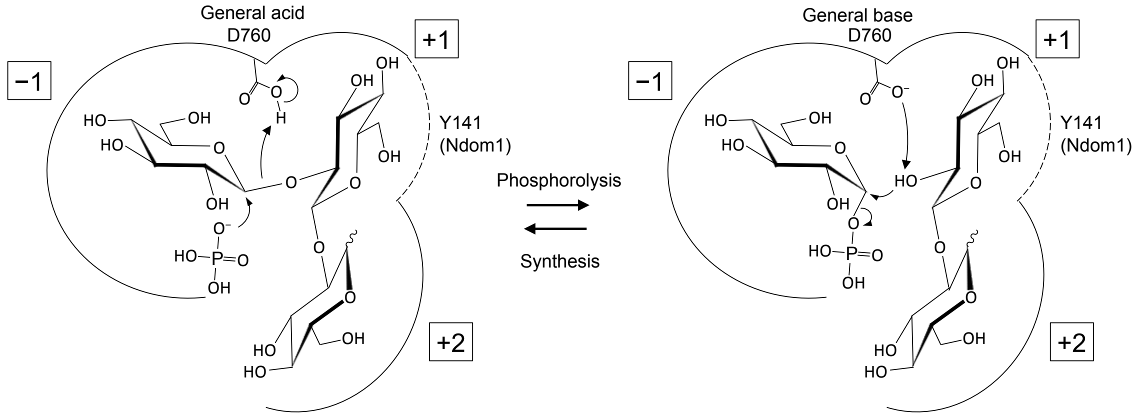

Figure S3. The proposed reaction mechanism of LpSOGP.

Subsites are shown in *boxed* numbers. *Dotted* lines represent that Ndom1 participates in constitution of a part of subsite +1. Sop_3 is drawn as one of the representative substrates.

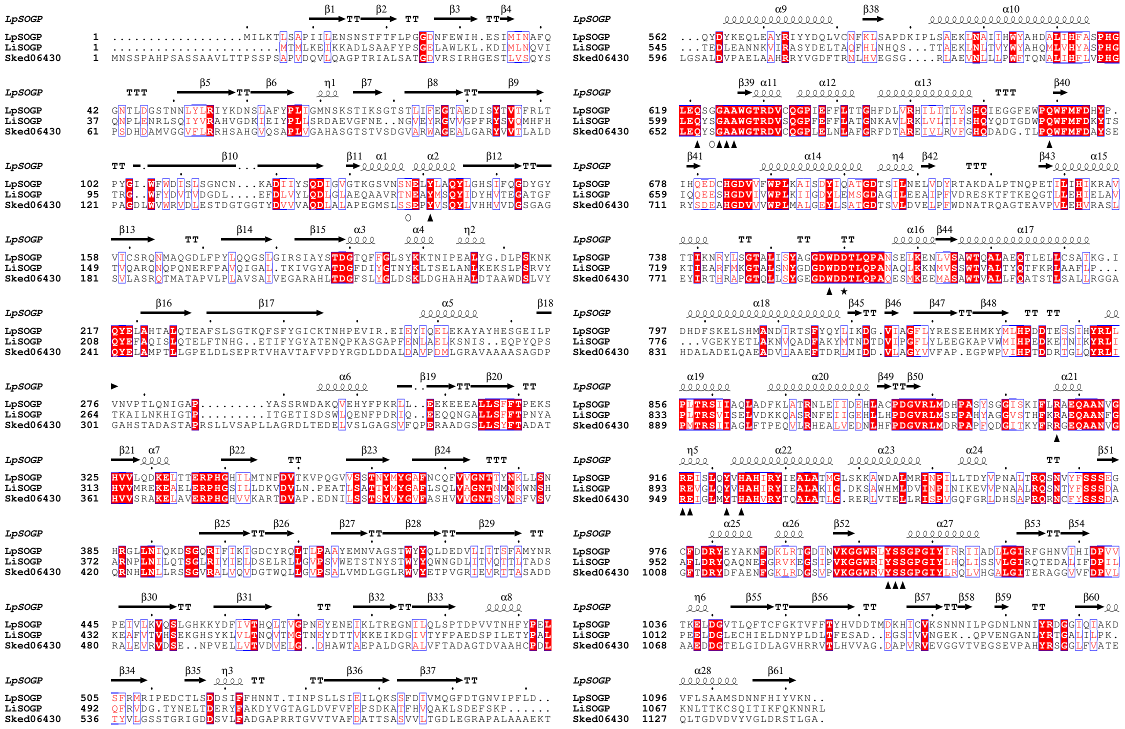

Figure S4. Multiple alignment of SOGPs and a SOGP homolog.

Multiple alignment was performed using ClustalW2 (http://www.ebi.ac.uk/Tools/msa/clustalo/). The figure was created using Espript 3.0 in the ENDscript server[54]. Catalytic acid residues are indicated by a *closed star*. Conserved and nonconserved residues related with substrate recognition are indicated by *closed triangles* and an *open circle*, respectively. The amino acid sequence identities between LpSOGP and LiSOGP, LpSOGP and Sked06430 from *Sanguibacter keddieii*, and LiSOGP and Sked06430 are 39%, 33%, and 34%, respectively, according to NCBI blast (http://blast.ncbi.nlm.nih.gov/Blast.cgi?PAGE=Proteins).
